# Progress in Ophthalmology

**Published:** 1898-04-16

**Authors:** 


### Progress in Ophthalmology.

(*Continued from page* 13.)

**Foreign Bodies.**—In reference to the localisation of foreign bodies in the eyeball by means of the Röntgen rays, by far the best method seems to be that demonstrated by Mr. McKenzie Davidson[1] at the January meeting of the Ophthalmological Society (and published in the *British Medical Journal*, February 5th, 1898). "The patient was seated upright, and his head fixed in a rectangular rest. The photographic plate was placed against the temple of the affected side behind cross-wires. A lead wire to act as pointer was made to touch the edge of the lower eyelid opposite a known point in the eye, and the patient fixed his gaze on some distant object during the exposure, in order that the eye might remain fixed. The exposures were made in the same way as for other parts of the body, and the interpretation of the skiagraphs was carried out by the use of his cross-thread localiser." This plan seemed much the simplest of any we have seen, but still was sufficiently complicated to lead one to believe that, unless in the hands of an expert who was working at the thing at the time, the results would not be very accurate.

Following on this demonstration, Mr. Treacher Collins showed four cases in which the rays had been used as a practical application; none of them were cases which could have been determined from the clinical appearances. Two of the chips were, after localisation, withdrawn by means of the electro-magnet, and the size and situation were found to correspond exactly to those indicated from the skiagraph. Three cases, too, of suspected foreign body were found to be free, and the diagnosis cleared up once for all.

**Opthalmoscopic Evidence of General Arterial Disease.**—Mr. Marcus Gunn,[2] in a very able paper which he read before the Opthalmological Society on the above-mentioned subject, described the appearance of the diseased arteries as part of a change in which the arteries of the body generally, and of the brain in particular, shared. The general reflex of the vessel was brighter, the central light sheath was bright, and the whole artery was a lighter colour than normal. This was due to a hyaline change in the arterial wall; as a consequence of this change the circulation in the veins was impeded, and in some cases the vein became invisible where crossed by an artery. As a further result of this venous obstruction an œdema of the retina was set up, the effects of which were to blur the details of the fundus. In some cases the size of the arteries was not uniform. The arteries were sometimes very tortuous. There was a loss of translucency in the arteries, so that where a vein passed behind an artery it could not be seen. On the other hand, where a vein covered an artery, the latter could be unduly seen through the vein, because of the thickening of the arterial coat, and the partial emptying of the vein where the two crossed each other. As a consequence of the hardening of the arteries, there was an interruption of the venous current, the vein was distended, and often hæmorrhages took place along its course. The change in the arteries was a change in their coats, an irregular thickening; with this there was loss of carrying power, and hence tortuosity. The changes in the veins were due to the obstruction to the flow of blood—the walls of vein and capillary underwent degeneration, hence arose the hæmorrhages. Into the question of ætiology he did not go. The changes, he said, usually occurred between 40 and 50, and if they were well marked at this age the prognosis was grave.

Among the patients in whom he found these changes he often found migraine, indigestion, or gout, and chronic alcoholism was a notorious cause. In some of the cases of hæmorrhagic glaucoma this affection of the vessels was the cause of the hæmorrhages. There was a close association between these changes and renal disease, but the vessels of the eye often showed the change before the kidney gave any evidence of it. As for hemiplegia, the author said he had examined at one time all the cases of that complaint in the National Hospital; in seven of these the arteries were normal, in ten they were affected, and in seven the changes were quite characteristic.

**Diseases of the Cornea.**—C. C. Caléb,[3] of Lahore, in writing on keratitis and corneal ulcer, says the origin of the hypopyon in corneal ulcer is interesting. If one were guided by the majority of English text-books, one could not resist the inference that in abscesses and ulcers of the cornea, with hypopyon, the latter was always caused by posterior perforation—in other words, that the collection of pus in the anterior chamber was merely the outcome of a mechanical or passive filling from the pus deposit in the cornea, but such was not the case. He states that the presence of purulent matter in the anterior chamber is due to direct immigration of pus corpuscles along the anastomosing canals of the corneal corpuscles to the spaces of the ligamentum pectinatum iridis, and thence into the anterior chamber, and that this immigration also explains the iritis, which is so invariable a concomitant of hypopyon, and from which is undoubtedly also derived, though necessarily in a secondary manner, part of the purulent matter of the hypopyon.

**Eserin in Corneal Ulcer.**—The same writer seems to be very fond of eserin in the treatment of spreading ulcers of the cornea, as he says[4]: "The drug *par excellence* which we rely upon for the purpose of curing extensive infective ulcers of the cornea, no matter how originating, is eserin, in combination with the hydrochlorate of cocain, the formula for the collyrium being ℞ eserini sulphat; cocainæ hydrochlor., āā, gr. ij.; aq. destillatæ, ℥j. Under the influence of these drops instilled into the eye three to six times daily large sloughing ulcers of the cornea, with or without hypopyon, heal as if by magic," and the resulting opacity is thinner and more circumscribed than with any other form of treatment. The power of eserin to reduce intra-ocular tension is well known. The pressure, as Meyer has remarked, "is one of the chief causes in keeping back the normal nutrition, and the reparation process in the cornea, hence the beneficial action of eserine in suppurative lesions of the cornea"; and furthermore, apart from this indirect influence, there can be no doubt that eserin acts in a very direct manner on the suppurative process itself by checking diapedesis on the one hand, and by promoting the absorption of purulent matter on the other by causing dilation of the ciliary vessels. As for its disadvantage, where a symptomatic iritis complicates the issue, he says: "No doubt eserine produces congestion of the ciliary body and iris, and if used alone would enhance the already existing iritis, and lead to troublesome synechiæ. Its combination with cocain, however, entirely does away with such a possibility, and, indeed, from this point of view, there is no reason against the intercurrent use of atropin when there is iritis present, say once a day, till the iritis has subsided." He proceeds to say that he has had such beautiful results in numberless cases that he is drawn to the irresistible conclusion that for all forms of progressive ulcers they are to be regarded as specific remedial agents, just as much as mercury and iodide of potassium are for syphilis.

[1] British Medical Journal, Feb. 5, 1898. [2] Lancet, March 26, 1898. [3] Indian Medical Record, Jan. 16, 1898. [4] Indian Medical Record, March 1, 1898.

